# CD70 as a target in cancer immunotherapy: advances, challenges, and future directions

**DOI:** 10.3389/fonc.2025.1609840

**Published:** 2025-08-15

**Authors:** Ruchen Wu, Junze Chen, Gang Wang, Lulu Han

**Affiliations:** ^1^ Cancer Institute, Xuzhou Medical University, Xuzhou, Jiangsu, China; ^2^ Center of Clinical Oncology, The Affiliated Hospital of Xuzhou Medical University, Xuzhou, Jiangsu, China; ^3^ Jiangsu Center for the Collaboration and Innovation of Cancer Biotherapy, Cancer Institute, Xuzhou Medical University, Xuzhou, Jiangsu, China

**Keywords:** CD70, cancer immunotherapy, CAR-T therapy, monoclonal antibodies, tumor microenvironment

## Abstract

Cancer immunotherapy represents a paradigm shift in oncology by leveraging the immune system to target tumors. The therapeutic efficacy of these approaches depends critically on identifying molecular targets that enhance treatment responses while limiting toxicity. CD70, a TNF family member, has emerged as a promising target due to its overexpression in hematologic malignancies and solid tumors contrasted with restricted expression in healthy tissues. This differential expression profile implies that CD70-directed therapies could achieve tumor-specific cytotoxicity with reduced off-target effects. Nevertheless, key challenges persist, including optimizing delivery systems and elucidating the immunological consequences of CD70 modulation. This review synthesizes recent progress in CD70-targeted immunotherapy, evaluating both its therapeutic potential and current constraints to guide future clinical translation.

## 1 Introduction

Cancer immunotherapy has redefined oncology by mobilizing the immune system against malignancies (1). Its clinical successes across diverse cancers highlight the need to identify molecular targets that maximize therapeutic benefit while mitigating adverse events. CD70, a TNF family protein, exemplifies such a target with emerging therapeutic relevance.

As a type II transmembrane protein, CD70 activates T and B cell proliferation and differentiation through CD27 engagement, thereby modulating immune responses (2). While constitutively suppressed in normal tissues, CD70 becomes aberrantly expressed in hematologic and solid tumors (2), positioning it as a compelling target for immunotherapy. Anti-CD70 monoclonal antibodies suppress tumor progression by augmenting immune-mediated cytotoxicity (3–5). Combinatorial regimens incorporating CD70-targeted agents exhibit synergistic effects with conventional therapies. CD70-directed CAR T cells display precise antitumor activity, while bispecific CAR T cells co-targeting CD70 and immune effectors amplify tumoricidal responses (6, 7).

This review critically assesses CD70’s role in cancer immunotherapy by analyzing its mechanistic contributions to tumorigenesis and surveying current targeting strategies. We evaluate the clinical promise and limitations of these approaches, underscoring CD70’s significance in advancing therapeutic innovation. A deeper understanding of CD70 biology may catalyze the development of precision immunotherapies to improve cancer outcomes.

## 2 Expression of CD70 and CD27

CD70, a TNF family member, functions as a type II transmembrane glycoprotein with a 50 kDa molecular weight that forms trimers. Under physiological conditions, CD70 expression occurs transiently in antigen-activated B and T cells, natural killer (NK) cells, and mature dendritic cells (DCs) (2). Hematological malignancies demonstrate co-expression of CD70 and CD27 in leukemia, B-cell lymphoma, multiple myeloma, and T-cell lymphoma (2). CD70 also appears in solid tumors such as renal cell carcinoma, nasopharyngeal carcinoma, glioblastoma, melanoma, and carcinomas of the lung, cervix, breast, ovary, and mesothelium (8–13).

CD27, a TNFR family member, is a 55 kDa type I transmembrane protein that forms dimers. Physiologically, CD27 localizes to naive T cells, memory B and T cells, and certain NK cell subsets (2). In hematological malignancies, tumor cells co-expressing CD27 and CD70 evade immune surveillance within the tumor microenvironment, thereby promoting disease progression (14, 15).

## 3 The CD70/CD27 axis

The CD70-CD27 axis functions through (CD272)^3^-(CD703)^2^ complex formation (16). Axis activation triggers extracellular CD27 cleavage, releasing soluble sCD27 fragments in both physiological and pathological contexts (14).

**Figure 1:**
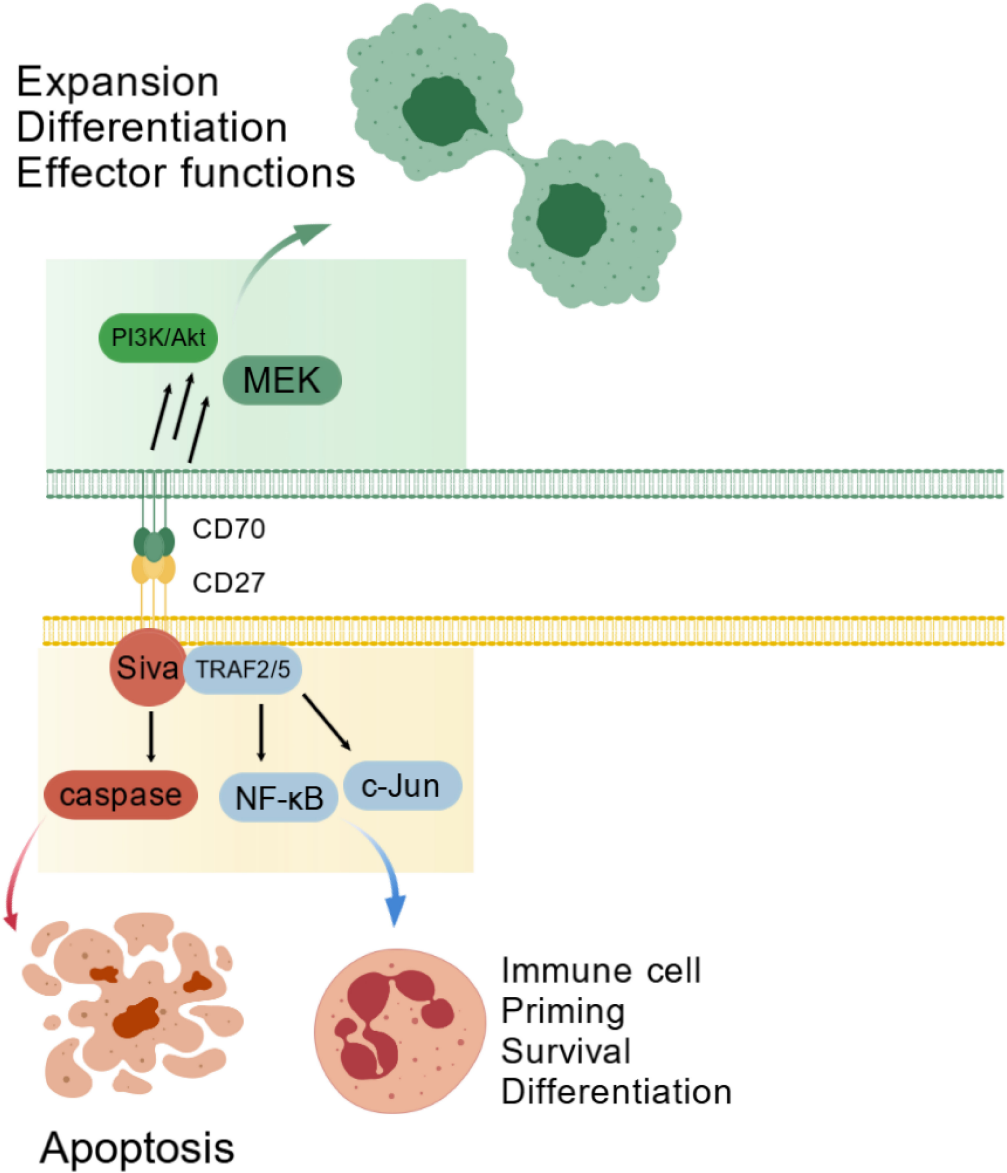
The CD70-CD27 axis in physiological immune regulation. The CD70-CD27 interaction plays a critical role in regulating immune cell proliferation, survival, and differentiation through TRAF2/5-mediated activation of the NF-κB and c-Jun signaling pathways. Conversely, this interaction may also trigger apoptosis via Siva-dependent caspase pathway activation. CD70 reverse signaling additionally stimulates the PI3K/Akt pathway, influencing cell survival, differentiation, and effector functions.

CD27 signaling engages TRAF2/5 to activate NF-κB and JNK pathways, driving T-cell proliferation, survival and differentiation. While promoting effector and memory T-cell development, this axis suppresses Th17 differentiation. CD70 signaling induces cytokine production in CD4^+^ and CD8^+^ T cells. Coquet et al. showed thymic CD27-CD70 interaction prevents regulatory T-cell (Treg) apoptosis, expanding the Treg pool (17). Paradoxically, the axis also triggers apoptosis via Siva and caspase proteins (18)(**Figure 1**).

**Figure 2:**
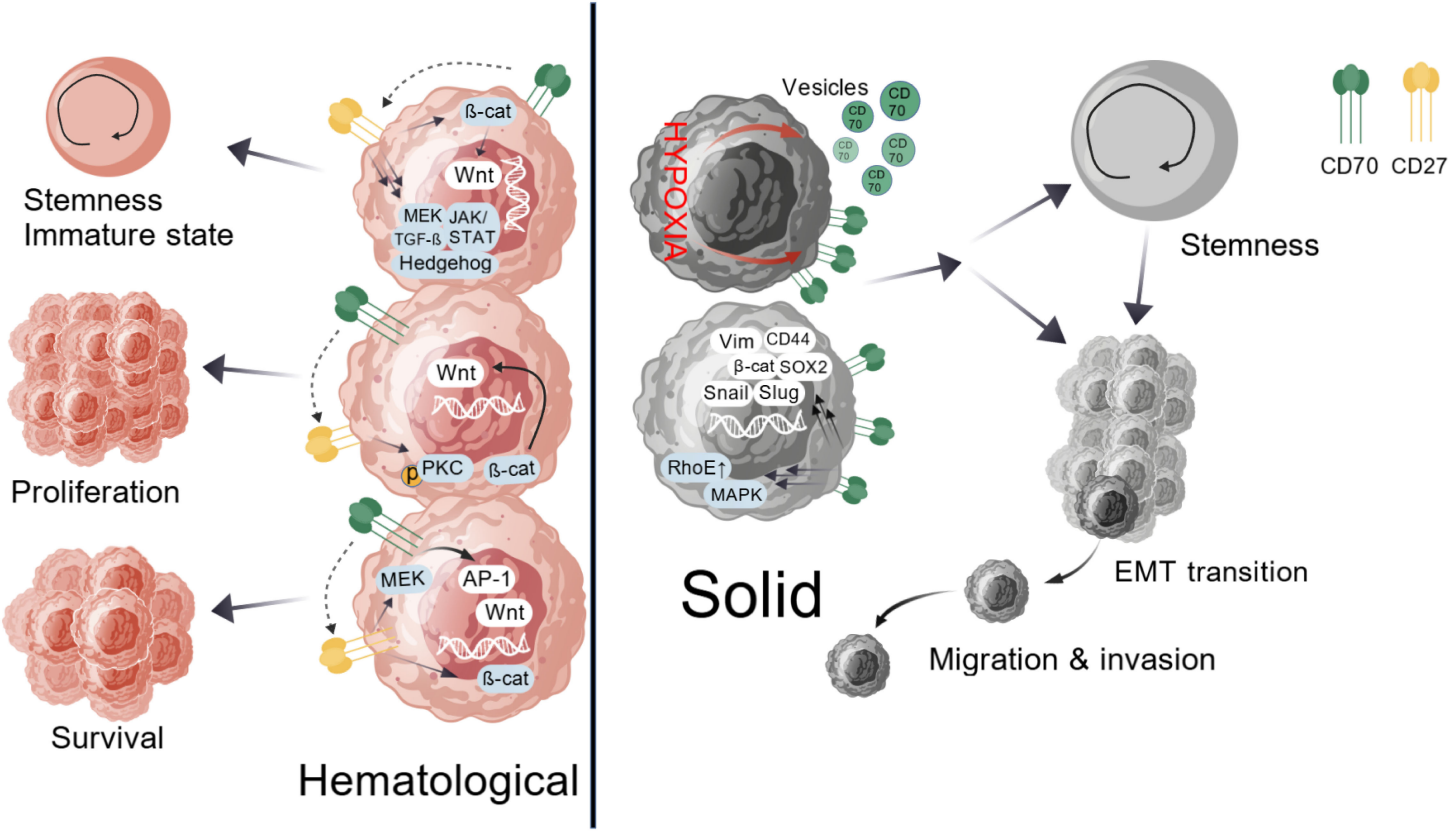
Pathogenic CD70-CD27 signaling in cancer. In hematological malignancies, CD70-CD27 signaling activates canonical Wnt, JAK/STAT, Hedgehog, and TGF-β pathways, which maintain cellular stemness and an immature phenotype. CD27 crosslinking via protein kinase C or β-catenin-dependent mechanisms, along with CD70 reverse signaling, drives malignant cell proliferation. This axis sustains survival through synergistic regulation of MEK pathway kinases and transcription factor AP-1, coupled with β-catenin-dependent Wnt pathway activation. Within solid tumors, CD70 signaling fosters cancer stem cell characteristics and epithelial-mesenchymal transition by upregulating EMT-associated transcriptional regulators such as SOX2, CD44, Vimentin, Snail, Slug, and β-catenin while concurrently activating MAPK signaling and inducing RhoE overexpression. Hypoxia emerges as a critical modulator of CD70 expression, directly influencing tumor stemness, migration, and invasive potential.

In solid tumors, CD70 signaling associates with cancer stemness and epithelial-mesenchymal transition (EMT) by upregulating EMT-related genes (SOX2, CD44, vimentin, Snail, Slug, β-catenin) while activating MAPK and overexpressing RhoE (2). This signal impairs T-cell function through three mechanisms: expanding suppressive Tregs, inducing T-cell exhaustion (evidenced by PD-1/TIM-3 expression in follicular B-cell lymphoma patients), and promoting apoptosis (2). Felix M Wensveen demonstrated that strong antigen stimulation activates CD95/CD95L through this axis, inducing T-cell apoptosis (19) (**Figure 2**).

The CD70-CD27 axis exhibits dual roles in hematopoiesis and oncogenesis. During normal development, tightly controlled CD70 expression supports immune cell priming and differentiation via TRAF2/5-dependent NF-κB and c-Jun activation (2), while Siva-mediated caspase activation can induce apoptosis (14). CD70 reverse signaling activates PI3K/Akt and MEK pathways, modulating cell expansion and effector functions (20) (**Figure 1**). In cancer, malignant cell co-expression creates autocrine proliferation loops. Hematologic malignancies show activated Wnt, JAK/STAT, Hedgehog, and TGF-β pathways that maintain stemness (21), with CD27 crosslinking and CD70 reverse signaling further driving proliferation (21). Survival pathways include MEK kinases, AP-1 activation, and Wnt/β-catenin signaling (22, 23). Solid tumors link CD70 to cancer stem cells and EMT (24) through EMT gene induction and MAPK/RhoE activation (2). Hypoxia potently regulates CD70 expression, enhancing stemness and invasiveness (25) (**Figure 2**).

This axis critically regulates immunity: NK cell CD70 activates CD27^+^ T cells to boost CTL responses and IFN-γ production, while DC CD70 sustains CD8^+^ T cell immunity (26). Germinal center B-cell interactions with CD27 modulate B-cell activation, plasma cell differentiation, and humoral responses (27).

## 4 Anti-tumor drugs targeting CD70

### 4.1 mAbs and ADCs

Monoclonal antibodies (mAbs) originate from a single B cell clone, producing a homogeneous population that binds to a specific antigenic epitope. Their mechanisms include antibody-dependent cellular cytotoxicity (ADCC), antibody-dependent cellular phagocytosis (ADCP), and complement-dependent cytotoxicity (CDC). By blocking CD70-CD27 interactions, mAbs may disrupt signaling pathways promoting blast proliferation and survival, potentially limiting immune evasion and augmenting antigen-specific T cell responses. Recent developments include anti-CD70 mAbs such as SEA-CD70.

Antibody-drug conjugates (ADCs) combine a monoclonal antibody with a cytotoxic payload via a linker, enabling targeted drug delivery. CD70-targeted ADCs like SGN-75 and MDX-1203 are under investigation for hematologic and solid tumors.

### 4.2 Anti-CD70 CAR-T

Unlike mAbs and ADCs, chimeric antigen receptor T-cell (CAR-T) immunotherapy functions as a dynamic immunotherapy, with engineered T cells capable of *in vivo* expansion and differentiation. This reduces dosing frequency while enhancing antigen recognition and tumor specificity. CAR-T cells also recruit immune cells through cytokine secretion, amplifying antitumor responses.

CAR-T technology merges antibody-like antigen targeting with T cell cytotoxicity by engineering chimeric antigen receptors. These receptors typically comprise an antigen-binding domain, hinge region, transmembrane domain, costimulatory domain, and signaling region (28). CD70’s tumor-selective expression makes it an attractive target. CD70-directed CAR-T has shown efficacy in acute myeloid leukemia, renal cancer, and glioblastoma.

#### 4.2.1 Anti-CD70 CAR-T and AML

Acute myeloid leukemia (AML) remains a therapeutic challenge, but CD70’s expression on leukemic blasts, not hematopoietic stem cells, positions it as a viable CAR-T target (29). Current CD70-targeted CAR-T approaches fall into two categories.

Tim Saucer et al. (29) engineered structurally diverse CD70 single-chain variable fragment CAR-T (CD70scFv CAR-T), demonstrating variable expression and antitumor activity. Wu et al. confirmed its antileukemic effects in preclinical models, though complete eradication proved unattainable (30). Despite their high binding affinity and specificity, scFvs present several limitations: Certain scFv constructs exhibit self-aggregation tendencies, which promote CAR clustering and subsequent tonic signaling that ultimately induces T-cell exhaustion. Additionally, murine-derived scFvs may trigger immunogenic responses.

The CD70-specific CAR incorporating truncated CD27 (tCD27) demonstrated superior functionality compared to conventional scFv-based CAR constructs. After Donald R. Shaffer et al. (31) pioneered full-length CD27-CD3 fusion constructs for B cell malignancies, Wang et al. (32) enhanced potency by truncating CD27’s intracellular domain and incorporating 4-1BB, though metalloproteinase-mediated CD27 cleavage impaired CD70 binding (33–36). However, CD27 engagement with CD70 triggers matrix metalloproteinase-mediated cleavage, which reduces functional CAR expression on T-cells and significantly impairs the *in vivo* efficacy of tCD27-based CAR-T therapy. Leick et al. (37) improved synapse affinity by pretreating with azacitidine to increase antigen density, while structural modifications to CAR domains mitigated metalloproteinase susceptibility.

To address this challenge, Cheng et al. (38) developed nanobody-based anti-CD70-CAR T-cells (nb70CAR-T) incorporating two distinct heavy-chain antibody variable domains (VHHs) for enhanced CD70 recognition. This design minimizes self-aggregation and immunogenicity compared to murine-derived scFv constructs. The researchers further employed CRISPR-Cas9 to disrupt the CD70 gene in T-cells, preventing fratricide. They also examined epigenetic modulators to control CD70 expression on AML cells, thereby optimizing nb70CAR-T efficacy.

#### 4.2.2 Anti-CD70 CAR-T and RCC

Renal cell carcinoma (RCC), a highly malignant tumor originating from the renal parenchyma and tubule epithelium, frequently overexpresses CD70 despite its role as a costimulatory immune molecule (39, 40). The rapid internalization of CD70 by antibodies enhances its therapeutic appeal in RCC (41, 42). ALLO-316, an allogeneic anti-CD70 CAR-T therapy, employs TALENs to disrupt TRAC and CD52 while incorporating a Rituximab recognition site to modulate T-cell overactivation via CD52 antibody and Fludarabine/Cyclophosphamide synergy. These allogeneic CAR-T designs circumvent GVHD risks while maintaining broad applicability. CD70^+^ T cells further correlate with a GvHD progression post-transplant, suggesting their suppression could mitigate this complication (43).

#### 4.2.3 Anti-CD70 CAR-T and glioma

Gliomas, the predominant primary brain tumors, originate from malignant glial cells. CD70 overexpression independently predicts poor survival in low-grade glioma (LGG) and glioblastoma (GBM) multiforme (44) and promotes macrophage infiltration and CD8^+^ T-cell apoptosis (10, 44), making it a compelling CAR-T target (10, 31, 32). Jin et al. linked CD70 to immunosuppression and augmented anti-tumor responses by engineering IL-8 receptor (CXCR1/CXCR2)-modified anti-CD70 CAR-T, leveraging radiation-induced IL-8 release to enhance tumor trafficking (45). Ji et al. enabled blood-brain barrier penetration by incorporating rabies virus glycoprotein 29 into anti-CD70 CAR-T (46), while Zhu et al. combined lysosomal virus infection with CAR-T to shift the microenvironment toward pro-inflammatory effector cell dominance (47).

### 4.3 CD70-CAR-NK

CD70 chimeric antigen receptor natural killer cells (CD70-CAR-NK), engineered with IL-15, hnCD16, and CD70 knockout, target tumor cells and cancer-associated fibroblasts with minimal off-tumor effects, exhibiting potent cytotoxicity and prolonged persistence (48). IL-15 stimulation amplifies CAR expression and cytokine secretion, improving tumor control in xenografts, particularly with repeated dosing (49). These cells also deplete alloreactive T cells, enhancing engraftment (50). Their off-the-shelf feasibility and dual targeting of tumor and stromal compartments address key CAR-T limitations, positioning them as versatile candidates for clinical translation.

### 4.4 Comparison of CD70-targeted therapeutic approaches

CD70-directed modalities exhibit distinct profiles: mAbs block CD70/CD27 interactions with low cytokine release syndrome risk but poor tumor penetration; ADCs deliver potent toxins yet face thrombocytopenia; CAR-Ts achieve durable responses despite fratricide and tumor microenvironment (TME) suppression; CAR-NKs offer GVHD-free, off-the-shelf therapy with stromal targeting but limited expansion. These trade-offs underscore the need for context-specific optimization (**Table 1**).

**Table 1:** Comparison of different CD70-targeted therapeutic approaches.

Therapy type	Mechanism	Strengths	Limitations/Challenges	Ref
mAbs	Block CD70/CD27 and ADCC/ADCP	Low CRS risk; Easy administration	Short half-life; Limited tumor penetration	(73)
ADCs	Drugs delivery via anti-CD70 antibody	High potency; Internalization advantage	Off-target toxicity (thrombocytopenia)	(74)
CAR-T	Engineered T-cell cytotoxicity	Tumor-specific; Durable responses	Fratricide; Cytokine release; TME suppression	(75)
CAR-NK	Off-the-shelf NK cell therapy	No GVHD; Targets CAFs; IL-15 enhanced persistence	Limited *in vivo* expansion data	(76)

The inherent heterogeneity of these therapies also manifests in their safety profiles, particularly the contrasting efficacy-toxicity trade-offs between CAR-T cells and ADCs. CAR-T therapy frequently induces CRS, while ADCs predominantly cause hematologic toxicities. CRS results from on-target immune activation, which often correlates with therapeutic efficacy in lymphomas but requires careful immunomodulation to control severe inflammation without eliminating CAR-T cells (51). In contrast, ADC-induced myelosuppression arises from off-target payload effects on bone marrow, lacking any therapeutic benefit and often forcing dose reductions that diminish antitumor activity (52). These distinct mechanisms necessitate different management strategies: CRS benefits from early cytokine blockade with agents like anti-IL-6, whereas ADC toxicities require supportive care and dose adjustments. Clinicians must balance CAR-T’s acute, potentially efficacy-associated toxicities against ADCs’ cumulative hematologic risks when selecting treatments.

### 4.5 Clinical landscape of CD70 therapies

Targeted therapies against CD70 have advanced considerably, though each modality presents unique clinical outcomes and limitations. **Table 2** summarizes ongoing and completed trials across mAb, ADC, and CAR-T modalities (**Table 2**). CD70-targeted therapies exhibit divergent clinical outcomes depending on therapeutic modality. The anti-CD70 monoclonal antibody cusatuzumab induced complete remission in 37% of acute myeloid leukemia patients when combined with azacitidine, with median overall survival reaching 11.5 months, but showed only stable disease in nasopharyngeal carcinoma before trial termination due to limited efficacy and adverse events including fatigue and pneumonia (NCT03030612). While cusatuzumab blocks CD70-CD27 interaction and mediates antibody-dependent cellular cytotoxicity, its efficacy appears restricted primarily to hematologic malignancies rather than solid tumors. Early-phase trials of SEA-CD70 suggest acceptable safety and preliminary antitumor activity, though comprehensive efficacy data remain unavailable.

**Table 2:** Comparison of different CD70-targeted therapeutic drugs in clinical trials.

Type	Number	Product	Mechanism	Cancer types	Phase	Trial status	ORR	DCR	Side effects
mAb	NCT04227847	SEA-CD70	A humanized, non-fucosylated mAb	AML, MDS	Phase I	Recruiting	NA	NA	Thrombocytopenia
mAb	NCT03030612	Cusatuzumab (ARGX-110)	A defucosylated IgG1 mAb	AML, High Risk MDS	Phase II	Completed	NA	NA	ThrombocytopeniaFatigue pneumonia
ADC	NCT01015911	SGN-75	Connect SGN-70 with monomethyl auristatin F	NHL, RCC	Phase I	Completed	NA	NHL:31.81%RCC:15%	Fatigue
ADC	NCT00944905	MDX-1203	Connect CD70- antibody with a small molecule drug MED-2460	NHL, RCC	Phase I	Completed	NA	NA	Fatigue
ADC	NCT02216890	SGN-CD70A	Connect SGN-70 with PBD	DLBCL, MCL	Phase I	Completed	20%	NA	Thrombocytopenia
CAR-T	NCT04502446	CTX-130	An allogeneic CD70 CAR-T product	T cell lymphoma, ccRCC	Phase I	Terminated	51.6%	81.3%	Cytokine release syndrome
CAR-T	NCT04696731	ALLO-316	An off-the-shelf, allogeneic CD70 CAR-T product	Advanced or metastatic ccRCC	Phase I	Recruiting	31%	NA	Neutropenia Thrombocytopenia

ADC, antibody-drug conjugate; AML, acute myeloid leukemia; ccRCC, clear cell renal cell carcinoma; DCR, disease control rate; DLBCL, diffuse large B cell lymphoma; mAb, monoclonal antibody; MCL, mantle cell lymphoma; MDS, myelodysplastic syndromes; NA, not available; NHL, non-Hodgkin lymphoma; NPC, nasopharyngeal carcinoma. ORR, objective response rate; PBD, pyrrolobenzodiazepine.

Among antibody-drug conjugates, SGN-75 demonstrated limited activity in RCC and non-Hodgkin lymphoma before development ceased following cases of immune-mediated thrombocytopenic purpura. The pyrrolobenzodiazepine-conjugated SGN-CD70A produced modest responses in diffuse large B-cell and mantle cell lymphomas but was constrained by dose-limiting thrombocytopenia occurring in 75% of patients. Although SGN-CD70A demonstrated a 78% disease control rate in metastatic RCC, its clinical development was ultimately constrained by marginal efficacy. MDX-1203 stabilized disease in 69% of patients but induced severe hypersensitivity reactions and delayed toxicities such as pleural effusions, prompting discontinuation (NCT00944905). These ADC toxicities predominantly involve hematologic complications, suggesting payload-related mechanisms despite CD70’s tumor-selective expression.

CAR-T cell therapies demonstrate comparatively robust clinical activity. The allogeneic CTX130 induced durable complete remissions in cRCC with an 81.3% disease control rate and manageable toxicity, leveraging direct T-cell cytotoxicity without ADC-associated payload effects. In T-cell malignancies, CTX130 yielded objective responses in 51.6% of patients at elevated doses. ALLO-316, another investigational CAR-T product, shows early promise in RCC. While CAR-T therapies enable sustained immune-mediated tumor clearance, they face challenges including cytokine release syndrome and complex manufacturing requirements.

Recent late-phase trials reveal both progress and limitations in emerging therapies. SGN-CD70A showed response rates of 20-30% but was hampered by severe thrombocytopenia (Grade ≥3: 40-50%), which constrained its clinical utility (NCT02216890). ALLO-316 produced durable responses (30-40% ORR) in CD70-high ccRCC patients, with certain remissions persisting beyond 12 months and a favorable safety profile characterized by mild CRS and negligible neurotoxicity (NCT04696731). The dose-limiting toxicity of SGN-CD70A contrasts with ALLO-316’s sustained activity, reinforcing CD70 as a viable target in solid tumors. Refining biomarker selection and CAR-T production protocols may improve therapeutic outcomes, suggesting allogeneic approaches could complement ADCs for CD70-positive malignancies.

Monoclonal antibodies and antibody-drug conjugates confront efficacy and toxicity barriers, whereas CAR-T therapies produce durable responses but require optimization to reduce immune-related adverse events and enhance production scalability. Future development should focus on engineered antibody-drug conjugates with refined linker-payload systems, combination strategies with immune checkpoint inhibitors, and improved allogeneic CAR-T platforms to broaden clinical applicability. These collective efforts affirm CD70’s therapeutic potential while emphasizing the need for modality-specific optimization.

## 5 CD70 and cancer diagnosis

Beyond its therapeutic potential, CD70 expression patterns also facilitate novel diagnostic applications. 18F-FDG PET/CT has been widely used in clinical diagnostics, but its effectiveness in ccRCC remains limited due to inconsistent glucose metabolism and reduced tracer uptake. The incorporation of CD70-targeted PET/CT imaging has improved diagnostic accuracy for ccRCC. A recent pilot clinical trial assessed two novel CD70-specific single-domain antibody tracers, (68Ga)Ga-NOTA-RCCB3 and (68Ga)Ga-NOTA-RCCB6, revealing that (68Ga)Ga-NOTA-RCCB6 immuno-PET/CT effectively identifies metastasis and postoperative recurrence in ccRCC (53). Further development of the CD70-targeted tracer (18F)RCCB6 has demonstrated its diagnostic precision in detecting ccRCC metastasis across preclinical models and human studies (54). In a ccRCC patient-derived xenograft model, (18F)RCCB6 exhibited substantial tumor-specific uptake (10.63% ± 1.21% ID/g), with CD70 blockade significantly reducing this signal (0.53% ± 0.04% ID/g; p = 0.002), confirming target engagement. Initial clinical assessment (NCT06148220) showed robust (18F)RCCB6 uptake in metastatic ccRCC lesions involving lung, pancreas, muscle, bone, and intracranial sites (54). Subsequent analysis of imaging data from 15 patients further validated the clinical utility of [18F]RCCB6 for postoperative surveillance and treatment response assessment (55). A current multicenter trial (NCT06680089) is systematically investigating the diagnostic and prognostic potential of (18F)RCCB6 immuno-PET/CT in metastatic ccRCC.

## 6 Discussion

CAR-T therapy has demonstrated remarkable success in treating hematologic malignancies, but its efficacy against solid tumors remains limited. The TME impedes CAR-T cell infiltration, promoting exhaustion and dysfunction (56), while tumor antigens often exhibit low specificity, high heterogeneity, and sparse expression (57). Overcoming these barriers requires innovative CAR-T designs tailored to the unique challenges of solid tumors.

### 6.1 The challenge of TME

The TME drives cancer progression and treatment resistance by harboring immunosuppressive cells such as MDSCs and Tregs, alongside inhibitory signals like PD-L1 and TGF-β (58). These factors collectively suppress CAR-T cell activity. Strategies to enhance efficacy include chemotherapy to deplete immunosuppressive populations and checkpoint blockade to reactivate T cell function. Such interventions may remodel the TME, improving CAR-T cell persistence and anti-tumor responses. Key strategies involve targeting the CXCR4/CXCL12 axis to enhance T-cell infiltration in immunologically cold tumors (59, 60); while JAK/STAT or PI3K inhibitors can suppress myeloid-derived immunosuppression by blocking TAM/MDSC function and their secretion of IL-10 and TGF-β (61); Local Treg depletion via anti-CD25 antibodies or immunotoxins reduces suppression of effector T cells (62–64); whereas IL-15 fusion proteins directly stimulate CD8 T cells and NK cells (65, 66); DNA-damaging agents or PARP inhibitors may further augment immunogenicity by increasing mutational burden and neoantigen presentation (67). These approaches require customization based on tumor-specific TME features—such as PDAC’s CAF barrier or GBM’s low mutational load—and should be combined strategically to maximize immune activation while minimizing autoimmune toxicity.

### 6.2 The challenge of tumor antigens

CD70 offers a promising target due to its tumor-restricted expression and minimal off-target effects. Disrupting the CD70-CD27 axis further curbs tumor growth. However, broader challenges persist: tumor antigens often lack specificity, exhibit heterogeneity, or are expressed at low densities (57).

#### 6.2.1 The challenge of low specificity

While tumor-specific antigens (TSAs) are ideal targets, their rarity necessitates reliance on tumor-associated antigens (TAAs) like CD70, which is overexpressed in malignancies but transiently in activated lymphocytes. This selectivity reduces off-target toxicity, yet CD70 expression on CAR-T cells risks fratricide (68). Gene editing to eliminate CD70, TCR, and MHC genes can prevent GVHD, while bispecific CAR-T cells or synNotch receptors may further mitigate fratricide. Compared with other TNF family targets, CD70 has highly heterogeneous expression, low off-target toxicity and high efficacy (**Table 3**).

**Table 3:** Comparison of other TNF family targets.

Molecule	Normal tissue expression	Tumor overexpression	Key toxicity	Ref
CD70	Transient (activated immune cells)	60-80% (RCC, AML, glioma)	Low-grade cytokine release syndrome	(2)
CD40	Constitutive (APCs, platelets)	40-60% (B-cell malignancies)	Thrombocytopenia, Hepatitis	(77)
CD137	Inducible (activated T/NK cells)	30-50% (lymphoma, melanoma)	Severe hepatitis	(78)

#### 6.2.2 The challenge of high heterogeneity

Antigen heterogeneity limits monospecific CAR-T therapies. Bispecific designs, such as B7-H3/CD70-targeting tanCAR-T cells (6) or CD19/CD70 dualCAR-T cells (7), broaden target recognition. Alternatively, BiTE-secreting CAR-T cells (69) or synNotch receptors (59) enhance efficacy against heterogeneous tumors without off-target effects.

#### 6.2.3 The challenge of low density

Low antigen density compromises CAR-T cell recognition. Epigenetic modulators like decitabine, chemotherapy, or radiotherapy can upregulate target expression. For instance, cisplatin elevates CD70 in NSCLC (8), while irradiation boosts CD70 in prostate cancer models (70). These approaches may sensitize tumors to CAR-T therapy.

### 6.3 Optimizing CAR-T design

Structural modifications to CAR-T have enhanced its efficacy and specificity through targeted engineering of key domains. Hinge, transmembrane, co-stimulatory, and signaling domains critically influence CAR-T functionality.

CD70-targeted CAR-T therapies have advanced through iterative generations. First-generation constructs, comprising anti-CD70 scFv linked to CD3ζ, induce T-cell activation but exhibit poor persistence due to lacking co-stimulation. Second-generation CARs incorporate CD28 or 4-1BB domains, with CD28 enhancing cytotoxicity and 4-1BB improving metabolic fitness and durability—key for mitigating fratricide from CD70’s expression on activated T-cells. Third-generation designs combine multiple co-stimulatory domains (e.g., CD28-4-1BB-CD3ζ) but risk excessive tonic signaling without clear preclinical superiority. Next-generation approaches integrate safety modules: (i) CRISPR-mediated CD70 knockout to prevent fratricide, (ii) cytokine-secreting armored CARs (e.g., IL-15) to counter immunosuppression, and (iii) logic-gated systems to reduce off-tumor toxicity. Persistent challenges include exhaustion from tonic signaling, antigen heterogeneity in solid tumors, and B-cell aplasia due to CD70 expression on plasma cells. Clinical trials are evaluating optimized second- and next-generation constructs.

### 6.4 CD70-targeted CAR-T therapy faces a critical challenge: fratricide

Fratricide occurs when CAR-T cells self-destruct through mutual recognition of surface-expressed CD70 (71). Mechanistically, this process involves scFv-mediated binding of CARs to CD70 on neighboring T cells (trans-interaction), which triggers cytotoxic activation (68). Beyond T-cell self-killing, CD70 sharing also drives off-target toxicity in vital tissues, particularly renal proximal tubular epithelial cells in RCC. Therapeutic targeting of these constitutively CD70-expressing cells—whether by ADCs or CAR-T-derived cytokines—induces direct cellular damage or cytotoxic payload internalization. Preclinical models and clinical ADC data demonstrate that RCC patients consequently develop dose-limiting acute kidney injury, characterized by elevated serum creatinine and proteinuria.

Two principal strategies have emerged to address fratricide and its downstream effects: (1) For scFv-based CARs, cis-masking prevents self-recognition while preserving tumor targeting, thereby eliminating trans-interaction-mediated fratricide and facilitating clinical translation (68). (2) In VHH-based nanoCAR platforms, CRISPR/Cas9-mediated CD70 knockout resolves both trans (fratricide) and cis interactions, the latter of which induces severe T-cell exhaustion through nanoCAR binding to CD70 on the same cell (72). Single-cell transcriptomics confirms this approach prevents exhaustion and restores antitumor efficacy in lymphoma PDX models, while simultaneously reducing renal toxicity by eliminating CD70 from T cells that could exacerbate off-target damage (72).

### 6.5 Evolution of CD70-targeted therapies

Advances in gene editing, AI, and delivery systems are addressing current limitations. CRISPR enables multiplexed edits (e.g., CD70/PD-1 knockout) and TME-responsive elements, while AI models predict resistance patterns from single-cell data. Delivery innovations like conditionally activated vectors and LNPs, combined with epigenetic modulators (e.g., azacitidine) or adenosine axis inhibitors, may overcome resistance. Key priorities include: (1) A global CD70 expression atlas, (2) Trials of allogeneic CD70-CAR-NK for solid tumors, (3) Companion diagnostics via PET tracers and computational modeling.

## 7 Conclusion

CD70, minimally expressed in normal tissues but upregulated in malignancies, regulates immune cell function physiologically yet promotes tumor progression. Current immunotherapies—mAbs, ADCs, and CAR-T—show promise, particularly in hematologic cancers. Challenges remain in overcoming TME suppression and antigen heterogeneity. Optimizing CAR-T design, enhancing tumor infiltration, and modulating the TME are critical to realizing CD70’s therapeutic potential.
